# Recurrent Indolent Keratitis Caused by Achromobacter xylosoxidans: A Case Report and Literature Review

**DOI:** 10.7759/cureus.75472

**Published:** 2024-12-10

**Authors:** Haitham F Sahawneh, Jimena Franco, Mohammad Abusamak, Jeremy Bartley

**Affiliations:** 1 Department of Ophthalmology and Visual Sciences, Case Western Reserve University School of Medicine, Cleveland, USA; 2 Department of Ophthalmology, University Hospitals Eye Institute, University Hospitals Cleveland Medical Center, Cleveland, USA; 3 Department of Ophthalmology, San Antonio Eye Center, San Antonio, USA; 4 Department of Special Surgery, Al-Balqa Applied University, Salt, JOR; 5 Department of Ophthalmology, Amman Eye Clinic, Amman, JOR; 6 Department of Ophthalmology, UT Southwestern Medical Center, Dallas, USA

**Keywords:** achromobacter xylosoxidans, fluoroquinolones, indolent corneal ulcers, infectious keratitis, radial keratotomy

## Abstract

An 83-year-old male with a history of radial keratotomy and laser-assisted in situ keratomileusis (LASIK) presented with symptoms of a non-resolving corneal ulcer in the right eye that had been present for five months. The patient was treated with antibacterial, antiviral, and antifungal medications over that period, with multiple recurrences that prompted referral to our tertiary center for management. Following a 48-hour cessation of all medications, a corneal biopsy was performed which grew *Achromobacter xylosoxidans*.

In conclusion, *Achromobacter xylosoxidans* remains a rare cause of keratitis but should be considered in patients with a slow-progressing disease. Risk factors include previous corneal surgeries or trauma, topical steroid use, and contact lens wear. Because *A. xylosoxidans* grows slowly, it can have a deep impact on the cornea. To help antibiotics get into the cornea properly, epithelial debridement may be helpful. It does not respond to standard empiric antibiotic therapy. Ceftazidime and fourth-generation fluoroquinolones are better choices for treatment.

## Introduction

*Achromobacter xylosoxidans* is a Gram-negative bacillus typically found in humid environments and has been identified as a cause of nosocomial infections [1,2]. Infection secondary to *A. xylosoxidans* generally arises in immunocompromised patients and much less commonly in immunocompetent patients [3]. Its role as a significant pathogen, however, may be underestimated, as confusion with other non-fermenting Gram-negative rods such as Pseudomonas species may occur [1]. While recognition of this pathogen’s role in ocular infections has been increasing over the past few years, the literature remains limited regarding risk factors, clinical features, and antibiotic susceptibility. Identifying these characteristics can help prevent delays in diagnosis and complications. We present a case of keratitis due to *A. xylosoxidans* infection in an immunocompetent patient with a recurrent indolent course and provide a review of the literature.

## Case presentation

An 83-year-old male with a history of radial keratotomy and laser-assisted in situ keratomileusis (LASIK) was referred to UT Southwestern Medical Center for management of a non-resolving corneal ulcer in the right eye. The patient’s symptoms of redness, pain, and blurred vision originally began five months prior to presentation. At that time, the patient reported a sudden onset of symptoms and decided to seek care with an outside ophthalmologist one day later. On evaluation, he was found to have a corneal ulcer with hypopyon and subsequently started on treatment with moxifloxacin every hour and prednisolone acetate every hour. The patient improved after a week of treatment and was tapered off his drops. Shortly after discontinuation of his medications the ulcer worsened, and the patient was referred to a different provider for further management.

On his next examination, the patient did not show any obvious infiltrate but had hypopyon present. He was diagnosed with viral keratouveitis and treated with oral valacyclovir 500 mg three times daily and prednisolone acetate four times daily. A few days after starting treatment, the patient’s examination worsened with infiltrates now present. A corneal culture was performed and the patient was started on fortified vancomycin and tobramycin every hour. The cultures were unable to identify the causative organism. The infection gradually improved on fortified antibiotics and the patient’s regimen was gradually tapered over the next three weeks. A week after discontinuation of the antibiotics, the infection recurred and the patient was referred to a new provider for further management.

Examination of the patient at this time revealed a corneal ulcer with deep infiltrates and hypopyon suspicious for fungal keratitis. He was started on topical amphotericin and natamycin every hour. On this regimen, the patient's symptoms continued to worsen and he was then referred to UT Southwestern Medical Center.

**Figure 1:**
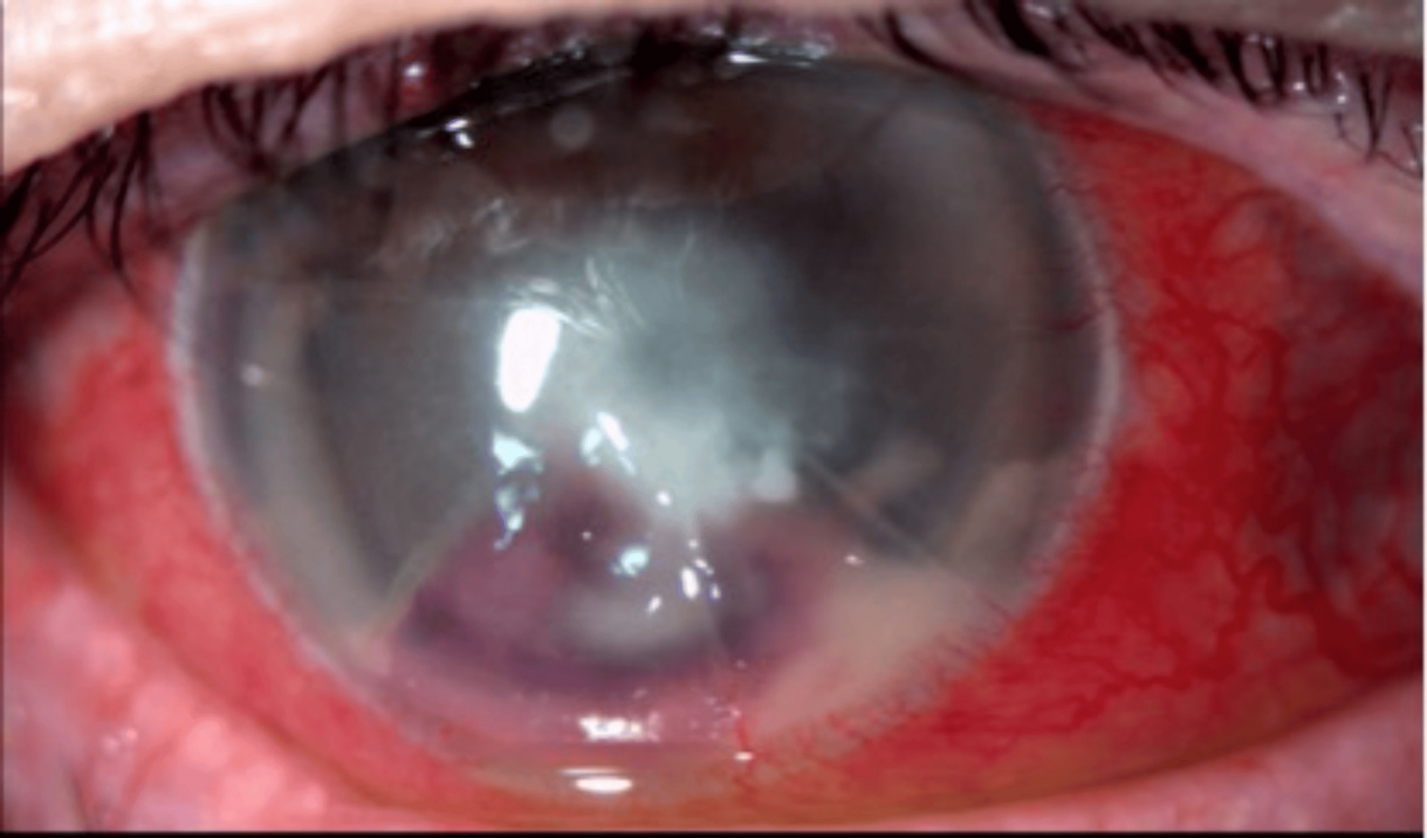
A deep, central, light blocking infiltrate with intact overlying epithelium and surrounding corneal edema.

The patient's medical history was remarkable for hypertension and hepatitis C. He had ocular history of radial keratotomy in both eyes in 1988 followed by LASIK of the right eye in 1995. The patient was pseudophakic and denied prior contact lens use. The vision of the right eye was found reduced to hand motion with reliable light projection. Sensation was found to be intact. The slit lamp examination showed an irregular, heaped epithelium with a deep, central 3.7 mm × 3.6 mm light-blocking infiltrate and surrounding corneal edema (Figure 1).

**Figure 2:**
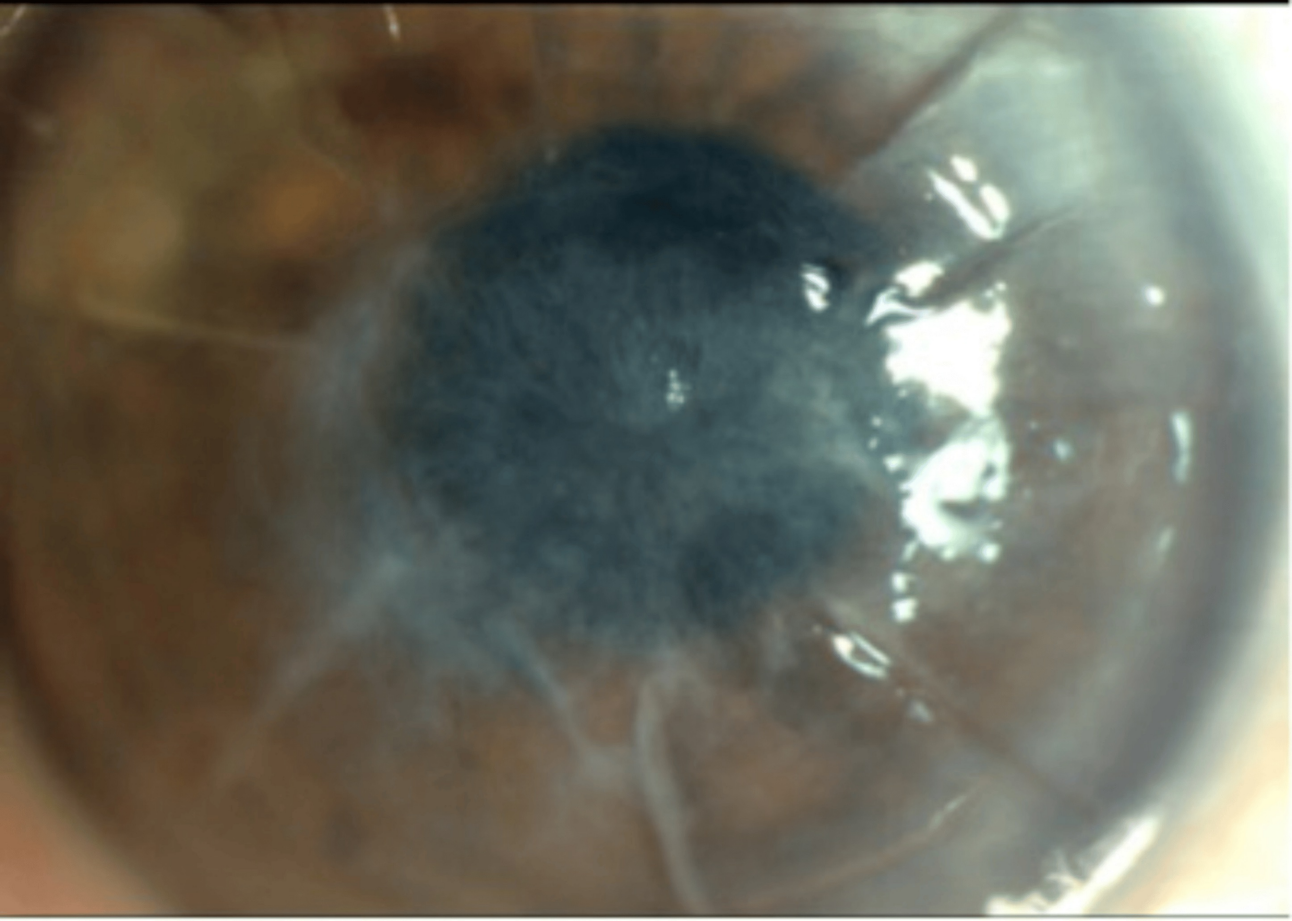
A resolution of infection with residual corneal scarring.

The infiltrate was noted to have increased density in the deep stroma and an endothelial plaque. The hypopyon was found to be mixed with blood. A confocal microscopy examination was performed during the clinic visit which was found to be inconclusive. All medications were stopped for 48 hours after which a corneal biopsy was performed. The patient was then started on moxifloxacin every two hours. Cultures grew *A. xylosoxidans* which was found to be sensitive to ceftazidime, cefepime, and piperacillin-tazobactam. There was intermediate sensitivity to amikacin, tobramycin, and gentamicin and resistance to aztreonam, ciprofloxacin, and tetracycline. The patient's examination had begun to show some initial improvement on moxifloxacin, so the medication was continued, and ceftazidime every two hours was also added. The patient continued to show improvement and antibiotics were tapered for four weeks (Figure 2). After two months of continued improvement, the patient underwent penetrating keratoplasty to help improve his vision.

## Discussion

*A. xylosoxidans* is a Gram-negative bacillus with a low incidence of infection in humans [4]. It is an opportunistic bacterium, with most infections occurring in immunocompromised hosts with an underlying disease [5]. Researchers have found it to be a rare but causative agent for ocular infections, specifically keratitis and endophthalmitis [6]. It is not easily identified and can often be mistaken for other Gram-negative rods, such as Pseudomonas species [1]. This may be due to similar motility and biofilm formation characteristics [4]. Researchers have used the morphology of flagella and antibiotic susceptibility profiles to differentiate between the two organisms [6]. Compared to Pseudomonas, *A. xylosoxidans* is less virulent and has a more insidious course, which may cause a delay in diagnosis, as was the case in our patient [2].

Previously described risk factors for the development of *A. xylosoxidans* keratitis include a history of penetrating keratoplasty, contact lens wear, and long-term use of corticosteroids [1,7]. Spierer et al. reported the largest case series of culture-positive infectious keratitis caused by *A. xylosoxidans* [7]. Of the 28 patients examined in this study, eight had previously undergone penetrating keratoplasty, and all but one patient was using topical corticosteroids. Eight patients also wore contact lenses. Reddy et al. conducted a separate study where they examined 10 patients infected with *A. xylosoxidans*, eight of whom developed keratitis [1]. Of eight patients with *A. xylosoxidans* keratitis, six had developed the infection after penetrating keratoplasty, two had a history of contact lens wear, and five had a history of topical steroid use. Our patient had no prior history of penetrating keratoplasty but instead had two prior refractive surgeries. Previous reports indicate that *A. xylosoxidans* infection occurs shortly after LASIK [8]. However, in our case, the refractive procedures had been performed 25 years prior to the infection, demonstrating that the risk of infection continues long-term. Throughout our patient’s treatment course, he reported intermittent topical steroid use. Our patient did not report a history of contact lenses.

Our patient's examination revealed a deep corneal infiltrate, which could potentially be secondary to the delayed presentation and slow progression of *A. xylosoxidans*. In our case, the initial moxifloxacin treatment regimen showed some improvement, but the presence of viable organisms likely persisted, potentially leading to premature treatment termination. The addition of topical steroids further deteriorated his presentation. Corticosteroids may depress delayed hypersensitivity mechanisms that protect against corneal infection by other Gram-negative rods, as well as have a similar effect on *A. xylosoxidans* [9]. Previous studies have noted similar presentations of deep corneal involvement with *A. xylosoxidans*, particularly in cases with delayed resolution [2,9,10]. We also noted an intact corneal epithelium in the present case, which could have contributed to the slowed resolution of the infection. Significant improvement was also noted shortly after the biopsy was performed, likely allowing adequate penetration of antibiotics.

The most frequent empiric treatment choices for bacterial keratitis consist of fluoroquinolones, and aminoglycosides may not be effective against *A. xylosoxidans* [7]. Previous reports have shown the antibiotic susceptibility of *A. xylosoxidans*. It has been found to be susceptible to extended-spectrum piperacillins, ceftazidime, amikacin, carbapenems, and trimethoprim-sulfamethoxazole [1,7,11]. Susceptibility to fluoroquinolones varies with increased sensitivity to fourth-generation fluoroquinolones and high resistance to ciprofloxacin [1,7]. *A. xylosoxidans* has also been found to have resistance to aminoglycosides such as gentamicin and tobramycin [12]. Our case showed a similar sensitivity profile as has previously been reported, with increased sensitivity to ceftazidime, meropenem, piperacillin-tazobactam, and trimethoprim-sulfamethoxazole. There was intermediate sensitivity to amikacin, tobramycin, gentamicin, and levofloxacin and resistance to ciprofloxacin. Sensitivity to moxifloxacin was unable to be obtained in our patient, but a good clinical response was noted, and it was presumed to be effective. The present case supports previous literature that recommends ceftazidime be considered the drug of choice for this microorganism [1].

## Conclusions

*A. xylosoxidans* remains a rare cause of keratitis but should be considered in patients with a slow-progressing disease. Risk factors include previous corneal surgeries or trauma, topical steroid use, and contact lens wear. Because *A. xylosoxidans* grows slowly, it may affect the cornea deeply. To help antibiotics get into the cornea properly, epithelial debridement may be helpful. It does not respond to standard empiric antibiotic therapy. Ceftazidime and fourth-generation fluoroquinolones are better choices for treatment.
